# Ontology-based systematical representation and drug class effect analysis of package insert-reported adverse events associated with cardiovascular drugs used in China

**DOI:** 10.1038/s41598-017-12580-4

**Published:** 2017-10-23

**Authors:** Liwei Wang, Mei Li, Jiangan Xie, Yuying Cao, Hongfang Liu, Yongqun He

**Affiliations:** 1 Department of Medical Informatics, School of Public Health, Jilin University, Changchun, 130021 China; 2 Department of Health Sciences Research, Mayo Clinic, Rochester, MN 55901 USA; 3 Library, Hebei Medical University, Shijiazhuang, Hebei Province 050017 China; 4 Unit for Laboratory Animal Medicine and Department of Microbiology and Immunology, University of Michigan Medical School, Ann Arbor, MI 48109 USA; 5 School of Bioinformatics, Chongqing University of Posts and Telecommunications, Chongqing, 400065 China; 6 Center for Computational Medicine and Bioinformatics, University of Michigan Medical School, Ann Arbor, MI 48109 USA

## Abstract

With increased usage of cardiovascular drugs (CVDs) for treating cardiovascular diseases, it is important to analyze CVD-associated adverse events (AEs). In this study, we systematically collected package insert-reported AEs associated with CVDs used in China, and developed and analyzed an Ontology of Cardiovascular Drug AEs (OCVDAE). Extending the Ontology of AEs (OAE) and NDF-RT, OCVDAE includes 194 CVDs, CVD ingredients, mechanisms of actions (MoAs), and CVD-associated 736 AEs. An AE-specific drug class effect is defined to exist when all the drugs (drug chemical ingredients or drug products) in a drug class are associated with an AE, which is formulated as a new proportional class level ratio (“PCR”) = 1. Our PCR-based heatmap analysis identified many class level drug effects on different AE classes such as behavioral and neurological AE and digestive system AE. Additional drug-AE correlation tests (i.e., class-level PRR, Chi-squared, and minimal case reports) were also modified and applied to further detect statistically significant drug class effects. Two drug ingredient classes and three CVD MoA classes were found to have statistically significant class effects on 13 AEs. For example, the CVD Active Transporter Interactions class (including reserpine, indapamide, digoxin, and deslanoside) has statistically significant class effect on anorexia and diarrhea AEs.

## Introduction

Worldwide, the incidence of cardiovascular diseases has been increasing in recent decades^1^. Coronary heart disease and stroke remain the two leading causes of death in the poorer regions of the world, including China, which will probably remain unchanged in 2020^2^, though the mortality from cardiovascular disease has declined in the US^3^ and most Western European countries^4–6^. In 1998, cardiovascular diseases claimed approximately 2.6 million lives, accounting for 40% of total deaths in China^7^. In 2014, cardiovascular diseases ranked the first major reason for death in China, accounting for 44.60% in rural areas, and 42.51% in cities^8^. The prevalence of cardiovascular diseases has been very high in the past decades. Consequently, the usage of cardiovascular drugs (CVDs), which are used to treat cardiovascular diseases, increased fast. A recent study revealed that Chinese hospital utilization of cardiovascular and cerebrovascular drugs increased 3.3-fold between 2006 and 2012^9^.

CVDs may lead to various adverse events (AEs) including serious adverse events (SAEs). In Dutch, 34% of all hospital admissions due to adverse drug events (ADEs) were associated with CVDs^10^. The rate of AEs induced by CVDs in Iran was 24.2%^1^. In 2013, 81.3% of suspected drugs responsible for ADEs in China were chemical compounds, of which 10% were drugs used for diseases of the cardiovascular system^11^. Using two-year clinical data from a tertiary care hospital in India, Palaniappan *et al*. identified 463 AEs from 397 patients, and 18.1% of the total AEs are related to CVDs^12^. In USA, FDA AE reporting system (FAERS) is the official drug-associated AE surveillance system^13^. FAERS has been used to mine AEs associated with CVDs such as statin^14^. However, no systematic study has been reported in analyzing AEs associated with large-scale CVDs using the FAERS database.

Biomedical ontologies with logical classification hierarchies have emerged and played important roles in knowledge management and data integration compared to vocabulary resources^15^. Specifically, a biomedical ontology is a human- and computer-interpretable set of terms and relations that represent entities in a specific biomedical domain and how these terms relate to each other. A biomedical ontology is computer-interpretable since the ontology is generated using a standard computer-understandable language such as the Web Ontology Language (OWL; https://www.w3.org/OWL/). A signature usage of ontology is the wide usage of the Gene Ontology (GO)^16^ to support gene expression data analyses. Since its first publication in 2000^16^, the original GO ontology has been cited by 18,000 publications. The Ontology for Biomedical Investigations (OBI)^17^, co-developed by over 20 biomedical communities, provides integrative representations of data in various areas of life-science and clinical investigations^18–21^. Other example usages of ontology include knowledge base construction^22^, data exchange^23^, natural language processing^24–29^, and metadata generation^20,30,31^.

The Ontology of Adverse Events (OAE) is a biomedical ontology designed from bottom to up to logically represent various AEs observed after medical interventions including drug administration^32^. Compared to controlled vocabulary terminologies such as the Medical Dictionary for Regulatory Activities (MedDRA)^33^ and the World Health Organization (WHO)’s Adverse Reaction Terminology (WHO-ART)^34^, OAE has many advantages such as the inclusion of textual definitions and references, logical axioms, and well-formed hierarchical structure^32^. Instead of definining an adverse event as an AE as shown in MedDRA and WHO-ART, OAE defines an AE as a bodily pathological process that occurs after a medical intervention and has an unintended outcome of a symptom, a sign, or a pathological process (e.g., infection)^32^. Such OAE definition logically links the AE with the medical intervention (e.g., drug administration), patient records, adverse health outcome, and temporal relation between the medical intervention and health outcome^32,35^. Empirical evidences^36–39^ show that OAE provides a more robust hierarchical structure defintions than MedDRA in terms of AE classification.

Developed by the Veterans Health Administration, the National Drug File - Reference Terminology (NDF-RT) uses a description logic-based, formal reference model that links drugs with active ingredients, groups ingredients into a hierarchy, and categorizes drugs and ingredients into classes of Mechanism of Actions (MoA)^40^. The combinatorial usage of OAE, and NDF-RT would allow the mapping and study of the relations among AEs, drugs, chemical elements, and drug mechanisms of action.

In pharmacovigilance, the term drug “class effect” was first used to describe the efficacy of beta-blockers in reducing total mortality for myocardial infarction^41^, and the term is now taken to mean the same or similar therapeutic or adverse effects of a “class” of drugs, *i.e*., drugs in the same group of chemical structures, mechanisms of action, or pharmacological effects^42^. To identify drug class effects, the levels of evidence from clinical experiments used to compare the efficacy and safety of drugs within the same class have been proposed^43^. Biomedical studies have found various beneficial class effects of drugs in terms of treatments. For example, beta-blockers exert a possible class effect in treating acute myocardial infarction^44^, and angiotensin converting enzyme-inhibitors (ACEIs) in treating heart failure patients^45^. It is also known that angiotensin receptor blockers have beneficial effects on glucose and lipid metabolism^46^. Adverse class effects of drugs also exist. A 2015 study uses visual and computational methods to explore the contribution of individual drugs to the class signal using MEDLINE literature data^47^. In this article, aligned with the previous definitions^43–45,47–49^, a class effect of drugs on a specific AE is also defined as an effect where all drugs in a defined class (or called category or group) are associated with the same AE. Identification of class effects of drugs on specific ADEs is important to drug development and patient treatment.

In this study, using drug package inserts and ontology-based technologies, we systematically studied the patterns of AEs associated with all available CVDs in China with a focus on drug class effect analysis.

## Methods

### CVD-specific AE data extraction

The names of active ingredients of all CVDs were first extracted from the Chinese textbook of New Pharmacology (17th version)^49^. Using these ingredient names, the database from the China Food and Drug Administration (CFDA) (http://app1.sfda.gov.cn/datasearch/face3/dir.html) was searched to identify the product names of the drugs that are manufactured domestically in China or imported abroad to China. The drug package insert documents were retrieved from the CFDA drug administration website (http://www.sda.gov.cn/WS01/CL1038/), or from drug manufacturers’ websites.

The CVDs, associated AEs, and other related information were initially collected using Microsoft Excel files. The section of “Adverse Reactions” of drug package insert documents contains text description of known AEs associated with the CVDs. These AEs were collected and inserted into an Excel file (see Supplemental File F1) with a pre-defined Excel template format. The Excel file also includes other information such as Chinese drug name, Chinese AE term, and English drug name.

### English translation of Chinese drug and AE names

English drug names of those CVDs are usually provided by Chinese drug package inserts. Manual English translation was conducted only for those drugs of which English names are unavailable in the package inserts. All Chinese AE names were also manually translated into English. To ensure correctness, all authors manually reviewed the Chinese and English translations until agreements were achieved.

### Ontology mapping and new OAE term addition

Translated English AE names were manually mapped to OAE terms. If there was no mapping, the new term was annotated and added into OAE by following the standard OAE term editing procedure^32^.

English drug names were mapped to NDF-RT (version 01/19/2012). Such mapping enables the query of drug products based on their active ingredient(s), ingredient classification, and the mechanisms of action (MoAs) for different drugs. Most drug names in NDF-RT contain dosage information. However, some cardiovascular drug package inserts do not contain drug dosage information. In this case, we mapped the drug dosage to the lowest dosage of the drug from NDF-RT.

### Development and query of OCVDAE

The Ontology of Cardiovascular Drug Adverse Events (OCVDAE) was developed using the format of W3C standard Web Ontology Language (OWL2) (http://www.w3.org/TR/owl-guide/). To develop OCVDAE, we first used OntoFox (http://ontofox.hegroup.org/) to extract two ontology subsets: (1) an OAE ontology subset that includes all the CVD-associated AE terms, and their related terms in OAE; and (2) a NDF-RT ontology subset that includes all the CVDs and associated ingredients and the mechanisms of actions (MoAs). Like OAE, OCVDAE is aligned with the upper level Basic Formal Ontology (BFO)^50^. For ontology consistency, the extracted NDF-RT terms were also aligned with the BFO structure. Specifically, the drug products and drug ingredients are aligned under BFO:material entity branch, and the MoAs are aligned under the BFO:role branch. After the two ontology subsets are aligned under OCVDAE, we used Ontorat (http://ontorat.hegroup.org/)^51^ to automatically generate annotations (e.g., Chinese drug names) and axioms that link drugs with AEs in OCVDAE. The input files of the Ontorat operation were the Excel files described above. The Ontorat output OWL files were then directly imported into OCVDAE. The overall ontology results were visualized and manually edited using a Protégé OWL editor.

The OCVDAE source code and related documents have been released to the OCVDAE GitHub website: https://github.com/OCVDAE. The ontology was deposited to the Ontobee website: http://www.ontobee.org/ontology/OCVDAE as well as BioPortal: http://purl.bioontology.org/ontology/OCVDAE.

The OCVDAE information is queriable using SPARQL^52^. The SPARQL queries can be performed under the Protégé OWL editor, or conducted using the Ontobee SPARQL web page after its deposition in the Ontobee RDF triple store^53^.

### Calculation of drug proportional class level ratio (PCR) for an AE

To calculate the class effect of a drug class on an AE, we designed and calculated a proportional class level ratio (PCR) of drugs in a drug class for a specific AE. Such a PCR score represents the ratio between the number of drugs in a class that are associated with an AE and the total number of drugs in the class. For a specific drug class and a specific AE, the PCR score is defined in Equation (1):

1 $$PCR\_for\_drug\_AE=\frac{No\_of\_drugs\_in\_a\_class\_associated\_with\_AE}{No\_of\_drugs\_in\_the\_class}$$

where the numerator is the total number of drugs associated with the AE in the drug class, and the denominator is the total number of drugs in the class. To count if a drug belongs to a drug class or not, mechanisms of action (MoA) hierarchy or related drug ingredient hierarchy as defined in NDF-RT can be utilized. All drugs under a specific ontological hierarchy of a class are considered as a drug under this class. Note that the specific AE can be a bottom level AE or intermediate or top level AE.

Our development of the PCR score is designed to mathematically calculate the drug “class effect”. Specifically, a drug “class effect” on AE is defined as a condition when all drugs in a class have the same AE^47^. Correspondingly, with the background of the total number of drugs from the same class, PCR calculates the percentage of drugs in the drug class associated with specific AE. Therefore, the drug class effect can be defined as the condition when PCR = 1. It is clear that PCR is critical to the analysis of drug class effect to drug AE. Furthermore, we used the PCR scores for heatmap analysis for better exploration of drug class effects on different AEs.

A drug can mean a drug chemical ingredient or a drug product. Drug chemical ingredient and drug product are different. A drug ingredient is typically matched to one or more drug products. In this study, the drug-AE class effect associations are defined at the ingredient level instead of drug product level. It means that an identified AE associated with an ingredient class occurs in at least one drug product containing the ingredient. For example, different dosage forms (e.g., tablet or lipid solution) of a drug may be associated with different AEs. As long as one dosage form (e.g., tablet) having the drug ingredient is associated with an AE, the ingredient of the drug is counted as a positive association with the AE. However, it is likely that the other dosage form (e.g., lipid solution) for the same drug is not associated with this AE. The study of the dosage form effect on the AE outcome is not within the scope of this study.

### PCR-based heatmap exploration of drug class effects

Heatmap analysis was performed using R 3.1.3 to explore the correlation between drug classes and various AE classes from OAE. The hierarchical drug and AE classes were identified using NDF-RT and OAE, respectively. The heatmap was created using n × m count matrix, the value of each cell in the matrix is the PCR corresponding to the specific drug class and AE class. In this way, PCR scores were used to cluster the drug classes and AE classes.

### Detection of statistically significant drug class effects on AEs

While a PCR of 1 meets the definition of class effect, such a class effect may not be statistically significant within background of all drugs and all AEs considered. To identify statistically significant drug class effects on AEs, we adopted and modified the traditional methods (e.g., PRR, χ^2^, and minimal case filtering) of defining specific drug-AE associations.

The proportional reporting ratio (PRR) is a major statistical method for detecting the associations between individual drugs and AEs^54^. In this study, we extended the PRR algorithm to detect the class effect between drug classes and AEs. Specifically, the drug class level PRR (C-PRR) for a specific AE was computed to measure if a class (or group) of drugs is more associated with the specific AE. A 2-by-2 contingency table was constructed for the C-PRR calculation (Table 1). The number of drugs with an AE and a drug class is defined as *a*. The number of drugs that belong to all other drug groups and are associated with the AE is assigned as *b*. The number of drugs belonging to the drug class, but having no association with the AE, is defined as *c*. The number of drugs unrelated to the AE or the drug class is defined as *d*. The C-PRR is then defined in equation (2):

2 $$C-PRR=\frac{a/(a+c)}{b/(b+d)}$$

**Table 1:** A two-by-two contingency table for C-PRR calculation.

	All drugs in a drug class	All drugs in all other drug groups
an AE	*a* (No. of drugs)	*b* (No. of drugs)
Not the AE	*c* (No. of drugs)	*d* (No. of drugs)
Total	*a* + *c*	*b* + *d*

A large C-PRR score of the AE indicates that the drug class-AE association is richly reported compared with other drug class-AE associations available in the database. In our study, all CVDs and all their associated AEs are our database for the calculation. In pharmacovigilance field, PRR usually uses a cutoff of 2^54^. The same cutoff can be used for C-PRR calculation.

Based on the same 2-by-2 contingency table, the Chi-squared score can be calculated and used as a criterion for AE significance test. The cutoff of Chi-squared test is usually >4, which is approximately of P-value of <0.05^54^. A similar method can be easily used to derive the drug class Chi-squared (C-χ^2^) score.

An additional filtering method commonly used in drug AE analysis is based on the minimal case reporting number^54^. Similarly, our class effect analysis includes a cutoff of at least 3 drug ingredients under a specific drug class category for a specific AE.

## Results

**Figure 1:**
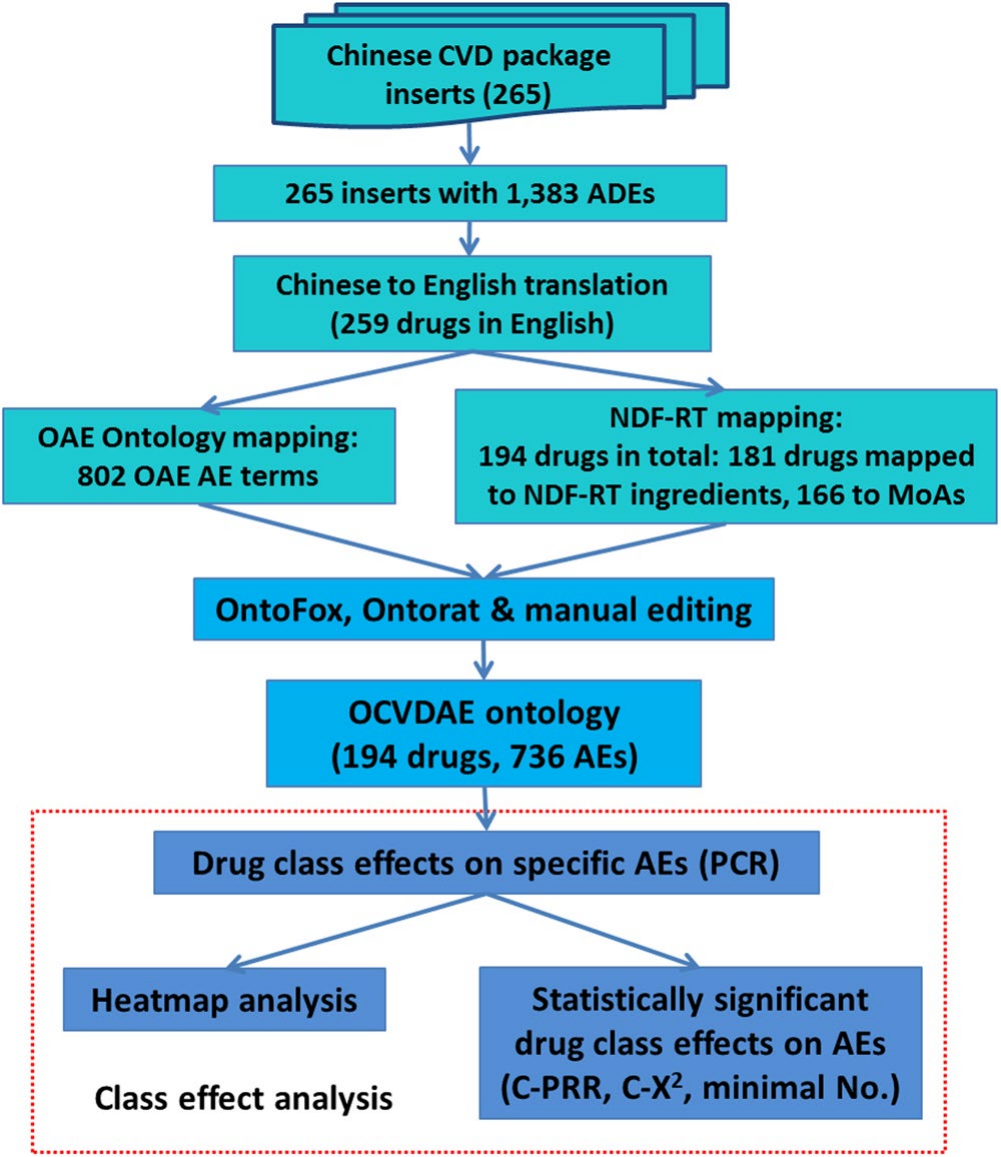
Workflow of our ontology-based cardiovascular drug class level effects on different adverse events (AEs). The results of collected CVDs, associated AEs, and ontology mappings are also labeled in this figure.

Overall, this project aimed to systematically collect, ontologically represent, and analyze chemical drugs used to treat cardiovascular diseases (i.e., CVDs) and their associated AEs recorded in package insert documents, and we have focused our analysis on drug class effects on AEs (Fig. 1).

### Collection and annotation of CVDs and their associated AEs

Our focus on the CVDs used in China market is because the health issue of cardiovascular diseases is very severe in China^2,7,8^, and the usage of various CVDs to treat cardiovascular diseases has dramatically increased^8,9^. The development of OCVDAE allowed us to seamlessly integrate different types of data, including CVDs, CVD ingredient classes and MoAs, and AEs, facilitating systematic queries and class effect analysis.

In total, 259 drugs used in China were identified to treat cardiovascular diseases. These CVDs were associated with 1,383 unique AEs in Chinese, corresponding to 802 unique English AE terms (see Supplemental File F1). One unique English AE term may include multiple Chinese translation names. Therefore, the number of the English terms is less than the number of Chinese terms. Among the 802 unique AE terms, 391 AEs already existed in OAE, and 411 AEs were added into OAE as new terms. In total 194 drugs can be mapped to NDF-RT, of which 181 drugs can be mapped to ingredients and associated classes, and 166 drugs can be mapped to MOA and associated classes. Supplemental File F1 is a master data Excel file that contains all the collected information of CVDs, NDF-RT terms and IDs of these CVDs, CVD-associated AEs, and OAE terms and IDs of these AEs.

**Figure 2:**
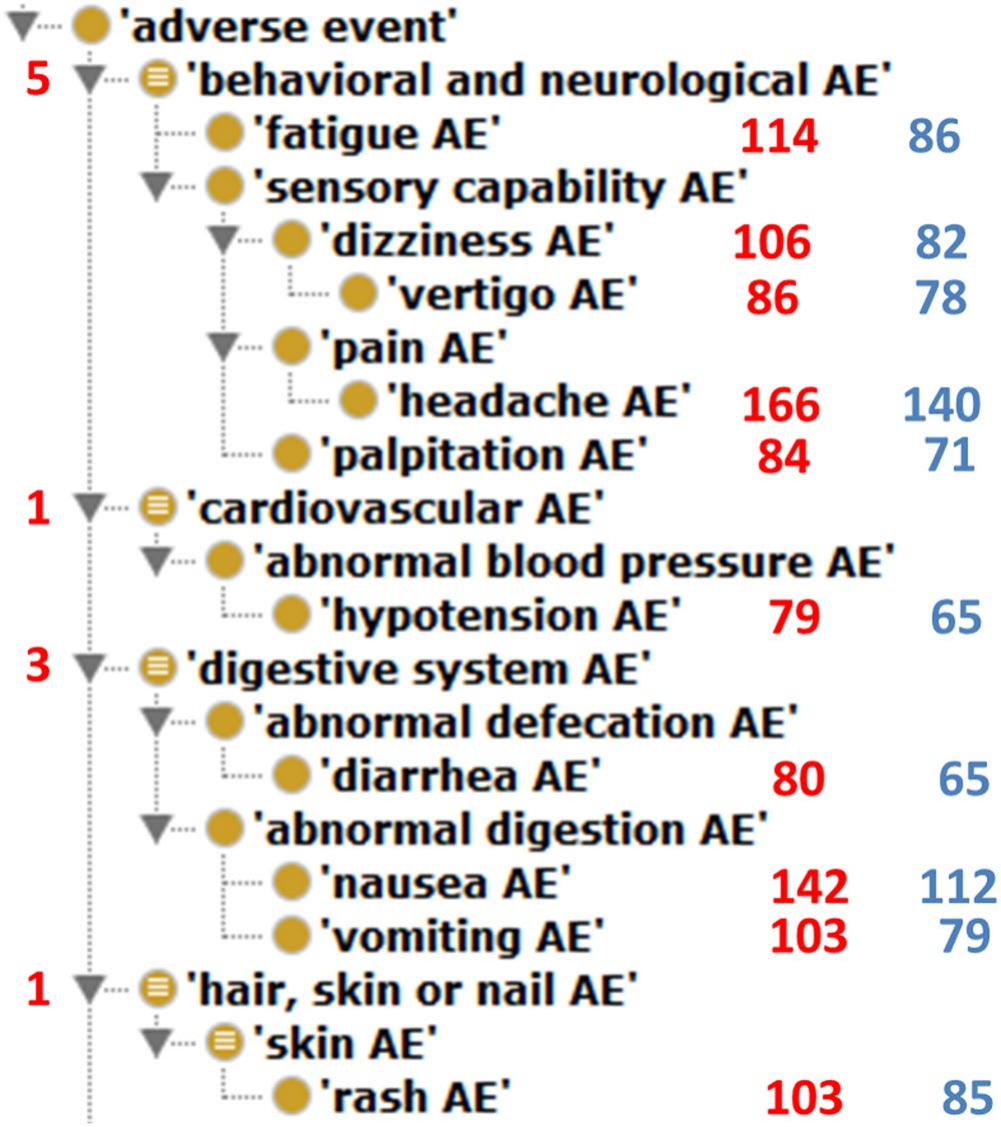
Classification of top 10 AEs associated with CVDs. These OAE terms are visualized using Protégé OWL editor. The left-side digits represent the numbers of specific AEs directly associated with CVDs. The right-side numbers represent the numbers of drugs associated with corresponding AEs. The red color-highlighted right-side numbers come from the total of 259 CVD drugs identified in our study. The blue color -highlighted numbers come from the total of 194 CVD drugs that were mapped to NDF-RT and included in the OCVDAE ontology.

To better understand the top AEs associated with licensed CVDs, the hierarchical structure of the top 10 AEs was extracted from OAE and visualized using the Protégé OWL editor (Fig. 2). Half of the top 10 most frequently reported AEs belong to behavioral and neurological AEs, followed by 3 digestive system AEs (i.e., diarrhea, nausea, and vomiting AE), one skin AE (e.g., rash), and one cardiovascular AE (i.e., hypotension). Note that hypotension raises alert for clinical use of CVDs in patients with the same condition.

### The OCVDAE: Systematic representation of CVD-specific AE information

The purpose of generating OCVDAE is to ontologically represent and seamlessly link all the information of CVDs, CVD ingredients, CVD mechanisms of action, and CVD-specific AEs at different hierarchical levels. The machine-readable OCVDAE can be reused and serve as a platform for further CVD-specific AE research.

**Figure 3:**
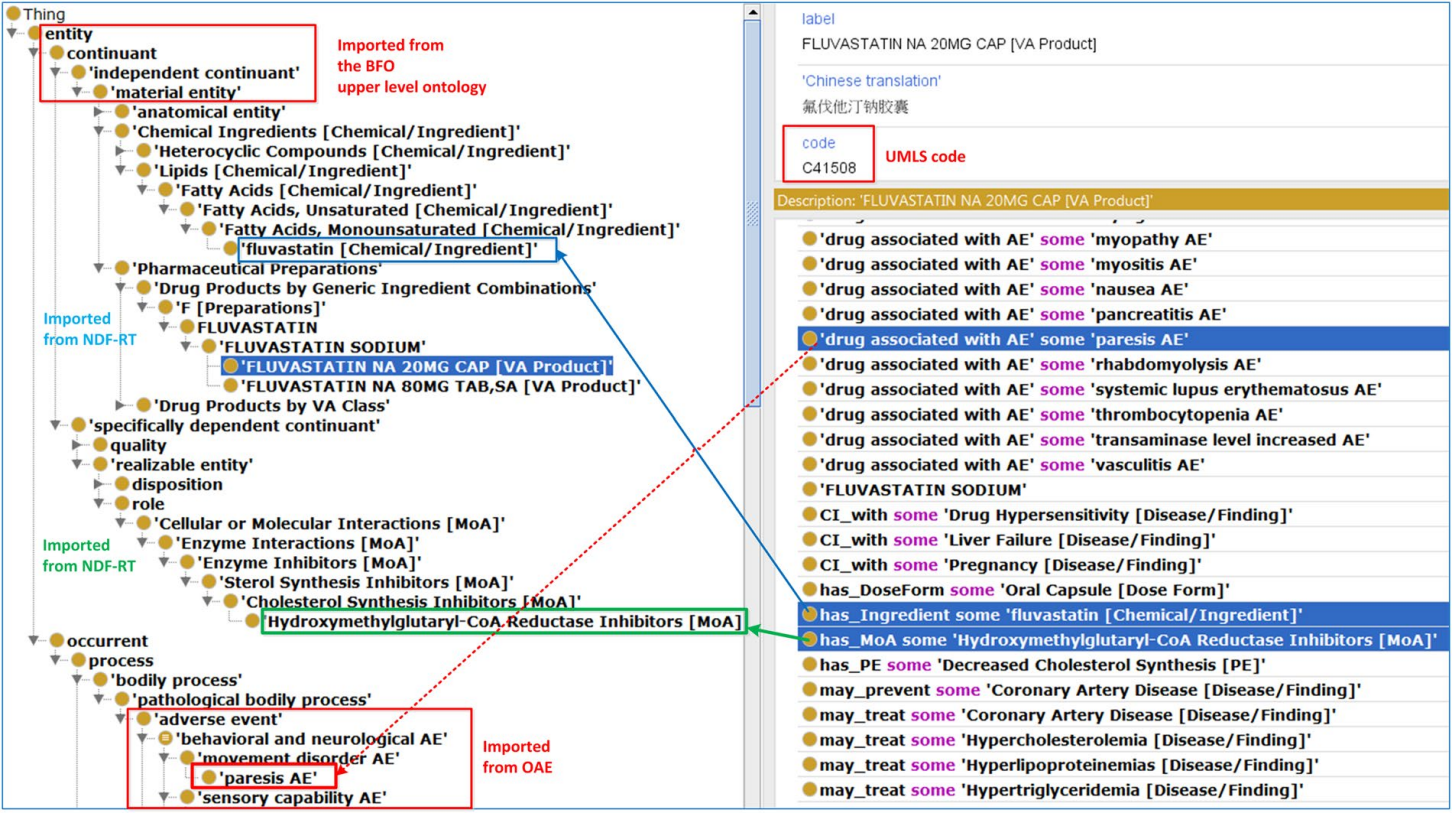
Integrative OCVDAE ontology representation of drugs, AEs, drug ingredients, and MoAs. This is a screenshot of Protégé OWL editor of a subset of OCVDAE after OntoFox extraction of two Fluvastatin drugs and their associated terms from OCVDAE. As shown in this figure, OCVDAE imports many terms from NDF-RT and OAE and uses the BFO as the upper level ontology. Each drug (e.g., Fluvastatin NA 20 MG CAP) is associated with its ingredient, MoA, and specific AE terms (e.g., paresis AE). These terms and their parent upper level terms are organized in a well-defined hierarchy in OCVDAE.

Formatted using the Web Ontology Language (OWL)^55^ format, our developed OCVDAE ontology includes 194 CVD classes, 2,948 classes and 126 annotation properties. It is noted that since some CVD classes mapped to more than one Chinese name, these 194 CVD drug classes were matched to 198 CVDs defined in Chinese. For these 194 CVD classes, OCVDAE includes corresponding CVD ingredients, MoAs, and associated 736 AEs (Fig. 1). To illustrate the hierarchical structure of OCVDAE, Fig. 3 was generated to show a subset of the OCVDAE that contains two fluvastatin drugs and their related terms, axioms, and hierarchies in OCVDAE. As seen in the figure, OCVDAE imports related terms from OAE^32^, and NDF-RT^40^. Like OAE, OCVDAE uses the Basic Formal Ontology (BFO)^50^ as the upper level ontology.

### OCVDAE knowledge queries and analysis

**Figure 4:**
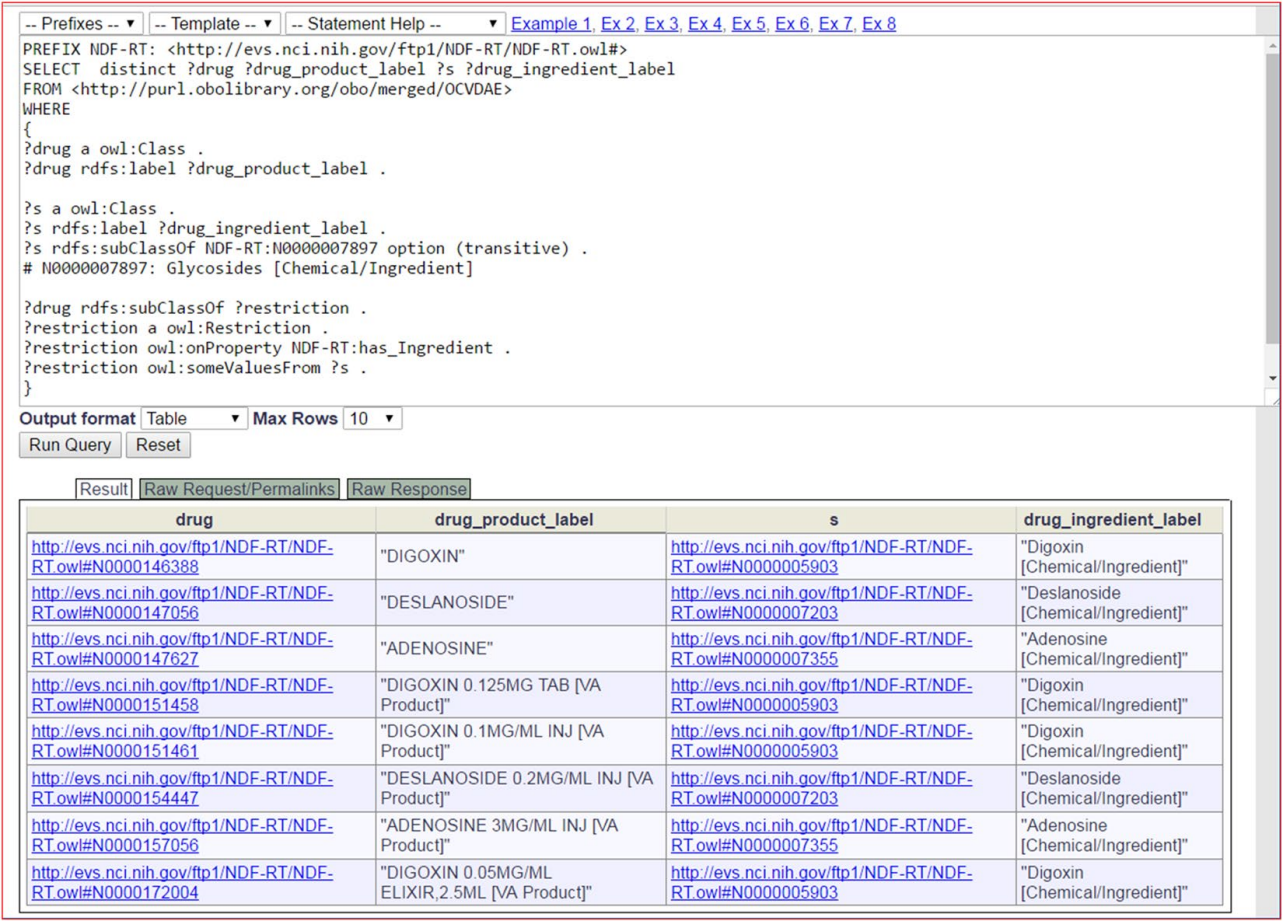
OCVDAE SPARQL script query example. The Ontobee SPARQL server^53^ was used to perform this query that identified drug-ingredient pairs with the condition of ingredients under the branch of NDF-RT “Glycosides [Chemical/Ingredient]” (N0000007897). This query returned 8 results, all related to digoxin, deslanoside, and adenosine.

The contents of the OCVDAE OWL file were expressed with Resource Description Framework (RDF) triples and stored in the Ontobee RDF triple store^53^. The RDF data model makes statements about resources in the form of subject-predicate-object expressions (*i.e*., triples). The SPARQL RDF query language^52^ is used to retrieve data stored in a RDF triple store. Figure 4 provides an example of querying the OCVDAE ontology using the Ontobee SPARQL query web page (http://www.ontobee.org/sparql)^53^. In this example, the SPARQL script was developed to query the chemical ingredients under the Glycosides class and their associated drug products (Fig. 4). Similar scripts can be developed to query other information in OCVDAE.

### Analysis of CVD drug class effects on AEs based on drug ingredient classification

**Figure 5:**
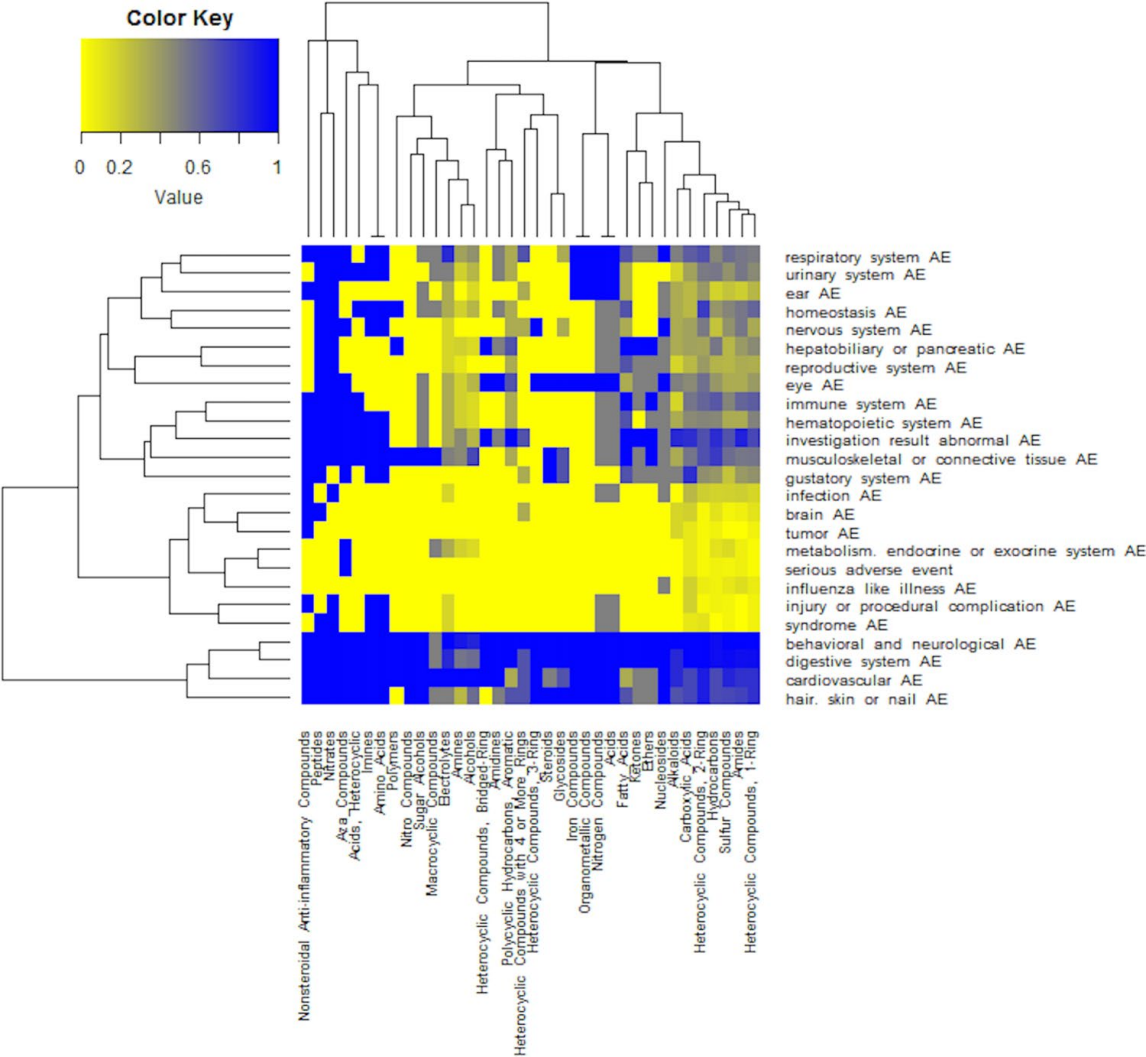
Heatmap analysis between 36 ingredient classes and AE classes based on PCR. Yellow, purple and blue are ordered from low to high number.

To illustrate our drug class effect analysis using drug ingredient classification, we generated the Fig. 5 heatmap that shows how 36 top level NDF-RT ingredient classes are related to AE classes based on the PCR scores. This level of 36 ingredient classes (e.g., “Fatty Acids”) is located at the third layer starting from “Chemical Ingredients” in NDF-RT (Fig. 3). Each of these 36 ingredient classes, e.g., Fatty Acids, includes its own hierarchy of chemical ingredients at different levels. A drug’s active ingredient (e.g., fluvastatin) usually is located at the bottom (or leaf) level of such a hierarchy (Fig. 3). Figure 3 also illustrates how our ontology represents the relation between a drug and its chemical ingredient component (or other pharmacological classes like MoAs).

These ingredient classes include 181 drugs used to treat cardiovascular diseases. The use of PCR scores in the heatmap allowed us to clearly visualize which drug class-AE associations represent the class effects. Specifically, since PCR = 1 indicates that the drug class effect, those cells with PCR = 1 represent class effects of drug classes on corresponding AEs or AE classes.

According to Fig. 5, 32 out of 36 ingredient classes have a class effect on behavioral and neurological AE, and 26 of 36 ingredient classes on digestive system AE. Many class effects on AEs are found from different ingredient classes, e.g., “Ethers” ingredient class and Electrolytes class (Fig. 5). Glycosides and other 12 ingredient class such as Iron Compounds and Peptides were found to have vision blurred AE.

**Figure 6:**
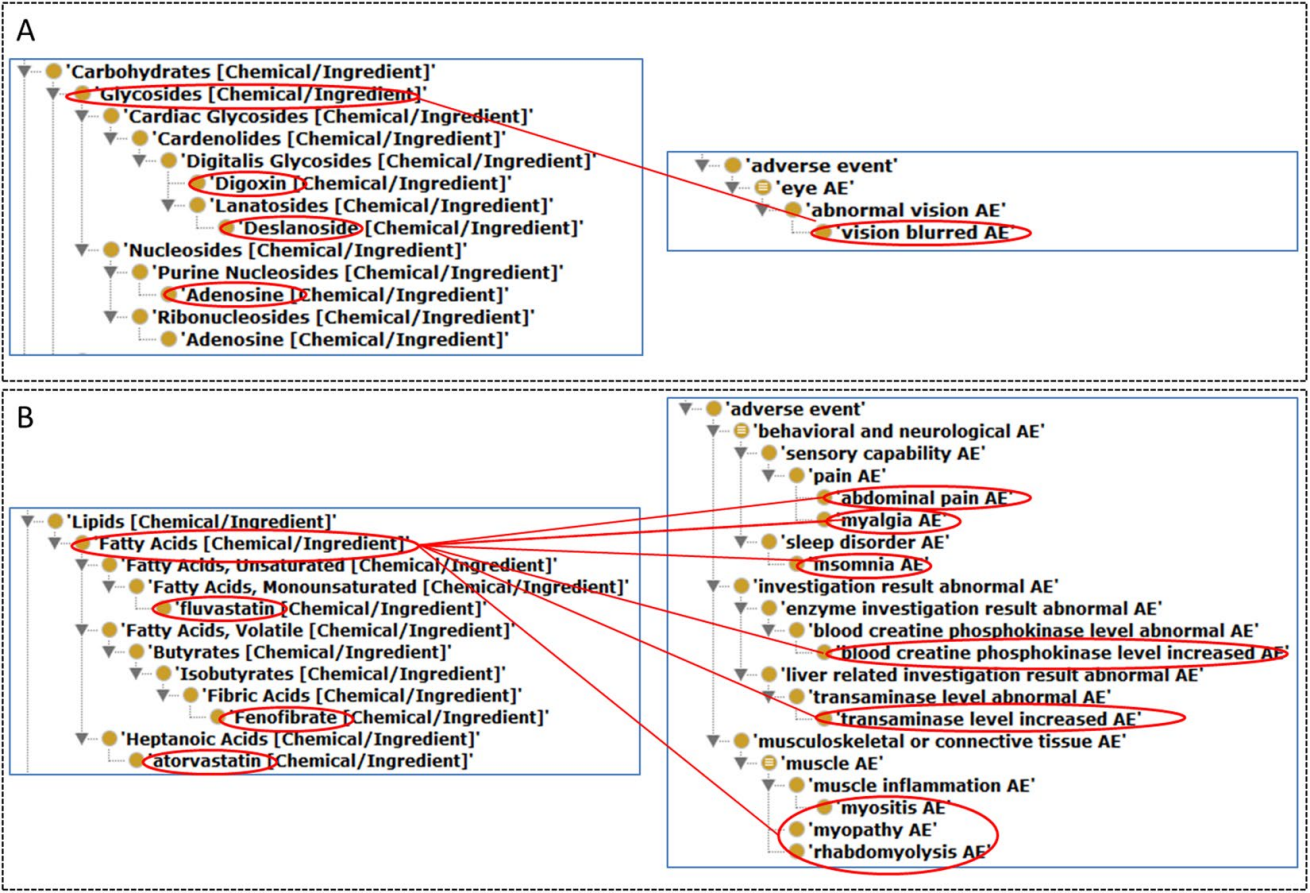
Statistically significant CVD class effects on different AEs based on 36 CVD ingredient classification. (**A**) Statistically significant class effect of glycosides on vision blurred AE, meaning that each of all three drugs under this class (i.e., adenosine, deslanoside, and digoxin) is statistically significantly associated with the AE. (**B**) Statistically significant class effects of fatty acids on 8 AEs. Only three drug ingredients (i.e., atorvastatin, fenofibrate, and fluvastatin) are placed under the fatty acids class in NDF-RT. Each of these ingredients is statistically significantly associated with each of these 8 AEs. See the text for more detail.

When the three additional criteria (i.e., C-PRR, Chi-squared, and drug number filtering) were used, only 2 of these 36 ingredient classes, i.e., Glycosides class (Fig. 6A) and Fatty Acids (Fig. 6B), were statistically significantly (Supplemental Table S1). As shown in Fig. 6A, Glycosides class (which has 3 ingredients: digoxin, deslanoside, and adenosine) has a class effect on vision blurred AE, which indicates that each of these 3 drug ingredients has an association with vision blurred AE. Furthermore, such a class effect is found to be statistically significant based on the three other factors: C-PRR, **χ** ^**2**^, and minimal drug number filtering.

Similarly, as shown in Fig. 6B and Supplemental Table S1, the fatty acids class has a class effect on 8 AE classes such as insomnia, abdominal pain, myopathy, myalgia, and myositis AEs. According to the NDF-RT, the fatty acid drug class of all the collected CVDs includes three ingredients: atorvastatin, fluvastatin, and fenofibrate (Fig. 6B). These three ingredients have been used for lowering lipids in patients.

### Analysis of CVD drug class effects on AEs based on drug MoA classification

Insights were gained by examining the association of NDF-RT mechanisms of action (MoA) with drugs. Specifically, our OCVDAE analysis identified a total of 26 NDF-RT MoA classes that are related to 166 CVDs (Supplemental Table S2). These 26 NDF-RT MoA classes are organized under the MoA class of ‘Cellular or Molecular Interactions’, which includes four direct subclasses: Biological Macromolecular Activity, Enzyme Interactions, Physiochemical Activity, and Receptors Interactions. Our study found that 97 CVDs are associated with Receptor Interactions, which further includes 8 drugs for active transporter interactions, 32 drugs for G-protein-linked receptor interactions, and 57 drugs for ion channel interactions. Among 33 CVDs for the Enzyme Interactions MoA class, 31 belong to the Enzyme inhibitors class, which includes 4 drugs for nucleic acid synthesis inhibitors, 1 for phosphodiesterase inhibitors, 11 for protease inhibitors, and 11 for sterol synthesis inhibitors (Supplemental Table S2).

Our MoA-based class effect analysis identified that 3 specific CVD MoA classes, each MoA class associated with different drug ingredients, have statistically significant class effects on 5 AEs (Table 2). Specifically, the Active Transporter Interactions MoA class has a class effect on anorexia and diarrhea AEs. The Active Transporter Interactions class includes 4 ingredients, i.e., reserpine, indapamide, digoxin, and deslanoside (Table 2). The Angiotensin-converting Enzyme Inhibitors class, which also includes 4 drug ingredients (e.g., enalapril) (Table 2), has a class effect on both cough increased and angioedema AEs (Table 2). In addition, the Cholesterol Synthesis Inhibitors class, which has 5 statin-related ingredients (Table 2), has a class effect on myalgia AE. It should be noted that the difference exists in that the class level effect is on specific class levels. For example, at the chemical structure level the CVD Lipids class includes three ingredients: atorvastatin, aluvastatin, and fenofibrate. While at the mechanism level, 5 statin-related ingredients (i.e., simvastatin, rosuvastatin, pravastatin, fluvastatin, and atorvastatin) are classified to have the same mechanism as Cholesterol Synthesis Inhibitors. NDF-RT classifies different drugs under these two categories. Different classifications may result in different results.

**Table 2:** CVD class effect based on CVD MOA classification.

MOA class and ingredients	AE	Ingredient No.	C-PRR	χ2	PCR
Active Transporter Interactions (reserpine, indapamide, digoxin, and deslanoside)	anorexia AE	4	3.05	7.28	1
	diarrhea AE	4	2.10	4.13	1
Cholesterol Synthesis Inhibitors (simvastatin, rosuvastatin, pravastatin, fluvastatin, and atorvastatin)	myalgia AE	5	4.00	12.19	1
Angiotensin-converting enzyme inhibitors (enalapril, captopril, benazepril, and perindopril)	cough increased AE	4	5.55	14.21	1
	angioedema AE	4	5.55	14.21	1

## Discussion

The contributions of this study are multiple. First, we for the first time systematically collected CVDs and annotated their corresponding AEs. Second, we developed an ontology OCVDAE to ontologically represent the CVD-AE information. Third, we developed and applied a combinatorial strategy to calculate statistically significant drug class-AE associations. Fourth, such a combinatorial strategy was applied to identify many scientific insights in terms of the AE patterns associated with CVDs at the drug class level.

To our best knowledge, our work represents the first study on collecting CVD-associated AEs from drug package insert information and ontologically representing and studying these AEs. Most studies on AEs of CVDs belong to retrospective studies without randomized and well-controlled clinical experiments, which primarily used clinical records of patients^56–58^, or belong to prospective case-series study that was based on information collected by interviewing patients, reviewing patients’ charts, laboratory test monitoring, and confirmation of physicians^59^. Existing studies mostly focused on AEs associated with specific classes of CVDs, i.e., cutaneous adverse reactions or suicide risk of calcium channel blockers^56,60^, or overall AEs of a specific class of CVDs, i.e., the retrospective evaluation of adverse drug reactions induced by antihypertensive treatment^58^. The systematic study of adverse reactions induced by CVDs at one hospital in Iran used prospective case-series method^59^. Different from these retrospective and prospective studies, our package insert data collection came from well-controlled randomized clinical trials, therefore providing accurate knowledge on drug-associated AEs. Meanwhile, exploratory studies are necessary in order to detect unknown drug AEs. Therefore, the evidences extracted from package insert documents and identified from spontaneously reported case reports or new clinical studies are complementary to each other.

OCVDAE is the first ontology targeted to represent AEs associated with drugs used to treat a category of diseases, i.e., cardiovascular diseases. Previously we have developed an Ontology of Drug Neuropathy Adverse Events (ODNAE), which ontologically represents all drugs leading to a specific category of AEs, i.e., neuropathy AEs^61^. Therefore, OCVDAE and ODNAE study drug AEs from two different aspects. It is noted that both OCVDAE and ODNAE extend OAE. To describe and organize liver injury induced by drugs, a drug-induced liver injury (DILI) ontology (DILIo)^62^. DILIo uses the Unified Medical Language System (UMLS) tool and the standardized terminology of the Systematized Nomenclature of Medicine-Clinical Terms (SNOMED CT). However, unlike OCVDAE or ODNAE, DILIo does not provide one-to-one associations between drugs and DILI histophological terms^62^. ADEpedia is a standardized knowledge base of AE using semantic web technology (38), which integrates general aspects of AEs and associated drugs. However, ADEpedia is not organized as an integrated ontology, which differs from our OCVDAE strategy.

The naturally hierarchical OCVDAE structure and knowledge provide an ideal framework for systematically analyzing the class effects of drugs on AEs. First of all, OCVDAE provides different hierarchical levels of class categorization of drugs, ingredients, and MoAs, and AEs. These hierarchical classes allow us to analyze class effects at different levels and with different class categorization. OCVDAE directly links drugs to AEs, allowing advanced queries and analyses. Second, we developed the mathematic PCR score to calculate class effects of drug classes on different AEs based on different drug hierarchies (e.g., ingredients, and MoAs). The PCR score is aligned with the general class effect definition, i.e., an effect of all drugs in a defined class having on the same AE. The PCR score provides a simple but powerful method to study class effects. For example, our PCR-based heatmap analysis allowed us to clearly visualize different class effects (Fig. 5).

In addition to the PCR-based class effect calculation and heatmap analysis, we further modified classical AE-drug association methods (i.e., C-PRR, C-χ^2^, and minimal number of drugs in a drug class) to identify statistically significant class effects. For the statistical drug class effect analysis, the background of C-PRR and C-χ^2^ is the total number of CVDs and all possible AEs associated with these CVDs with different drug classes. The C-PRR/C-χ^2^ methods test whether the specific AE is significantly associated with the specific drug class compared to all other AEs and all other drug classes in the database. We also use a minimal number of drugs in a drug class as a cutoff, which reflects the traditional method of using minimal case reporting number filtering for defining specific drug-AE association^54^. Such a combinatorial method provides a feasible strategy for detecting statistically significant drug class effect for specific AEs.

Our study differs in many ways from another study of drug class effect analysis using MEDLINE literature data and heatmap data analysis^47^. In that study, Winnenburg *et al*. extracted drug-AE pairs from MEDLINE by aggregating drugs into the Anatomical Therapeutic Chemical (ATC) classes and drug AEs into high-level MeSH terms. The association between drugs and AEs at the drug class level was explored using heatmaps and k-means clustering based on the PRR values that specified the significance of AE-drug associations^47^. In comparison, our study differs in data source, standard terminology, and method to identify class effect. Instead of using the MEDLINE literature, we used drug package insert data. Our semantic system used OAE for AE terms/hierarchy and NDF-RT for drugs, drug ingredients, and MoA classes. For the heatmap and clustering analysis, the biggest difference between these two methods is that our method uses the PCR while their method uses PRR. Since PRR does not include the information of drug classification, the PRR-based heatmap and k-means clustering analysis could only approximately and indirectly explore class effects (which rely on drug classification). Instead of using PRR values, we directly used the PCR scores that provide a simple and accurate method for directly calculating drug class effects on AEs based on drug ingredient or MoA classifications. Furthermore, we calculated and applied class level PRR (i.e., C-PRR), Chi-square (i.e., C-χ^2^) and minimal number filtering to generate statistically significant drug class effects on different AEs and AE classes.

In addition to the PRR and Chi-square methods used in this study for drug-AE association analysis, there are also other reported statistical methods, including the Bayesian Confidence Propagation Neural Network (BCPNN)^63,64^, Gamma Poisson Shrinkage (GPS)^65^, Reporting Odds Ratio (ROR)^66^, Reporting Fisher’s Exact Test (RFET)^67^. An R package PhViD^68^ is also developed to include these methods. All these methods are developed for general drug-AE association studies. In comparison, our PCR method is designed specificially for drug class effect analysis. The PCR value of 1 indicates a class effect result. Since PCR does not test statistical significance, the combined useage of PCR and statistical methods (e.g., PRR and Chi-square in this study) will allow the detection of statistical significant drug class effects.

Our findings showed that CVDs are associated with cardiac disorder AE significantly. Hypotension AE and palpitation AE are among the top 10 most frequently reported AEs (Fig. 2). Previous studies also revealed the potential of having hypotension AE following treatment with cardiovascular drugs, e.g., verapamil (a drug to treat hypertension)^69,70^. Nifedipine, a calcium antagonistic drug for treating high blood pressure, was also associated with short lasting palpitation event without arrhythmias^71^. Our findings confirmed the alert of potential risks of cardiac disorder AEs after the administration of CVDs.

Our OCVDAE-based drug class effect analysis identified many insightful drug class effects on different levels of AEs based on the drug ingredient or MoA classifications. For example, our statistically significant class effect analysis found that out of 36 CVD ingredient classes, the Glycosides class and Fatty Acids class was found to have class effects on one and eight AEs, respectively (Fig. 6). Three Glycosides class ingredients (i.e., digoxin, deslanoside, and adenosine) are all associated with blurred eye AE. The association between deslanoside (or adenosine) and blurred eye AE has not been reported in the literature; however, digoxin has been reported to be associated with blurred eye AE in peer-reviewed publications^72,73^. In such a class level analysis, drug classes were based on NDF-RT hierarchical definition, and AE or AE classes were derived from the OAE ontology.

Our statistical analysis found three MoA drug classes having class effects on different AEs (Table 2). Our MoA-based study found that the 4 drug ingredients under the MoA class of angiotensin-converting enzyme inhibitors have a class effect on increased cough and angioedema AEs (Table 2). Interestingly, a 2013 report has clearly indicated that angioedema and cough are the two most important adverse effects of angiotensin-converting enzyme inhibitors^74^. In addition, our study found that 5 statin-related ingredients (i.e., simvastatin, rosuvastatin, pravastatin, fluvastatin, and atorvastatin), which have the mechanism as Cholesterol Synthesis Inhibitors, all have the class effect on myalgia AE. Our result is consistent with many literature reports. For example, it has been reported that statin-associated muscle symptoms, which most often consist of myalgia, may occur in more than 10% of patients using statins^75,76^. Muscle toxicity caused by the statin has been focused, such as myalgia, myopathy and rhabdomyolysis^77–79^. Furthermore, myalgia is found to be the most common adverse event of statin use according to the review of MEDLINE database English articles^80^. While the mechanism by which statins produce muscle injuries is still unclear, reduction of the cholesterol content of skeletal muscle membrane is considered as a possible mechanism^81,82^. The other possible mechanisms include mitochondrial mechanisms^83^ and inhibition of farnesyl pyrophosphate (an intermediate for the production of ubiquinone (Coenzyme Q10))^81^. All these mechanisms are likely interconnected. For example, coenzyme Q is a member of the electron transport system of the inner mitochondrial membrane where it converts the energy in carbohydrates and fatty acids into ATP to drive cellular machinery and synthesis^84^, and treatment with Coenzyme Q10 significantly affects cholesterol production^85^. A recent report indicates that Coenzyme Q10 protects against statin-induced myotoxicity in zebrafish larvae^86^. How these individual mechanisms are interconnected to cause statin-induced myotoxicity deserves further investigations.

Our study also found that the Active Transporter Interactions drug class (with 4 ingredients) have a class effect on anorexia and diarrhea AEs (Table 2). The Active Transporter Interactions drug class includes 4 drug chemical ingredients: reserpine, indapamide, deslanoside, and digoxin. The finding of such a class effect on anorexia and diarrhea AEs means that each of the 4 chemical ingredients in the drug ingredient class has been found to be associated with these two AEs according to the package insert data we have collected. Such a finding suggests that some active transporter interaction(s), triggered by these drug ingredients, might participate in the formation of anorexia and diarrhea AEs. For example, reserpine is able to bind to the catecholamine transport system of synaptic vesicles and deplete catecholamines from peripheral sympathetic nerve endings^87^. Catecholamines regulate appetite control which is closely related to anorexia^88^. Deslanoside inhibits the Na-K-ATPase membrane pump, resulting in an increase in intracellular sodium and calcium concentrations^89^. The imbalance of electrolytes (including sodium and calcium) may result in anorexia. It has been found that anorectic patients have altered erythrocyte Na-K-ATPase pump^90^. Metabolic turnover of Na-K-ATPase may be regulated by catecholamines and other hormones^91^. Indapamide is an inhibitor of the the Na-Cl co-transporter (NCCT). The chloride depletion can cause electrolyte perturbations, hypokalemic alkalosis, and anorexia^92^. While digoxin-induced anorexia mechanism is unclear, digoxin-induced toxicity (including anorexia) is often elevated when digoxin is combined with other drugs (e.g., quinidine)^89^. Digoxin is a substrate of P-glycoprotein, an efflux transporter found throughout the epithelial cells of the intestine. It was found that quinidine inhibits the P-glycoprotein in the intestine and at sites of digoxin elimination (e.g., kidney)^89^. Therefore, digoxin-induced anorexia is often due to a drug-drug interaction that affects digoxin elimination and absorption through the P-glycoprotein efflux transporter system. Therefore, these four chemical ingredients (i.e., reserpine, indapamide, deslanoside, and digoxin) all interact with some transporters, which might lead to anorexia AE. By integrating the possible anorexia formation mechanisms induced by different drugs as described above, we hypothesize that these drugs induce anorexia by interacting with some components of an integrative transporter-mediated pathway, which likely includes a chain of transporter-mediated interactions that regulate the metabolic levels and activities of catecholamine and electrolytes. However, one drug may interact with an off-target receptor or transporter, and there might be other mechanisms of these drugs that contribute to the anorexia AE. Therefore, more experimental investigations are required to test this hypothesis and the role of the drug-transporter interactions in anorexia AE formation.

Our innovative technique revealed drug class effects based on the structural level as shown in the Fig. 5 heatmap and Mechanisms of actions level (Table 2). In our studies, it appears that the classification based on the Mechanisms of actions is more useful to generate plausible hypotheses, for example, our hypothesis about the Active Transporter Interactions drugs that have a class effect on anorexia and diarrhea. Meanwhile, our structure-based classification was only focused on the top two level structure classes. It is possible that class effects based on more specific and bioactive structural classification may become more useful, which can be further explored in the future.

Our study represents the first ontology-based methodology that identifies drug class effects on adverse events. Most existing drug class effect studies focus on the drug therapeutic effects. Since no systematical reference textbook or electronic resource is available to introduce drug class effects on certain adverse events, our study also provides the first source of summarized drug class effects on adverse events.

In our future work, we may examine the effects of different variables (e.g., age, gender, and drug dose form) on the class effect outcomes^93^. These variables may change the health outcomes and the classification of drug class effects on AEs. For example, a Korean study, analyzed the patterns of ADEs in different age groups and showed that CVDs, among other drugs, was reported more frequently as causative drugs for ADEs in the elderly^94^. Clear sex differences between women and men (54% vs. 46%, respectively) were seen in a Dutch study in hospital admissions for ADEs due to CVDs^10^. In addition, different drug forms (e.g., capsule vs tablet) may induce different AEs. A detailed examination of these variables to drug class effects will be very important for precision medicine.

In the future, we will also use our OCVDAE ontology as a platform to further add updated information to explain the basic mechanisms of CVD-associated AEs. For example, a new publication by Imbrici-2017 *et al*.^95^ discloses ClC-K channels as a novel target of the AT1 receptor blockers valsartan and olmesartan. The ion Channel Interactions [MoA] in OCVDAE does not mention the ClC-K chloride channels. To explain the AEs of valsartan and olmesartan by this mechanism of action, we can improve OCVDAE and include the reported reference.

Our current study focused on the PCR score of 1, which represents a class effect, i.e., all drugs in the drug class being associated with an AE. In the future, we may also examine the scenario with a PCR score less than 1, which indicates that only a portion of the drugs in a drug class are associated with a corresponding AE. The portion of the drugs may have its special meaning under different conditions; for example, this portion of drugs may be classified into a more specific drug subclass. The higher the PCR score, the more likely the drugs in a drug class are associated with a corresponding AE or AE class.

In this study, we focused our analysis of package insert documents of the CVDs used in China market. Our future work may also focus on the systematic analysis of class effects of different CVDs or other drug classes used in USA and European markets using package insert documents or other types of drug AE related data. The knowledge obtained will be added to OCVDAE. We will also periodically update OCVDAE when new information of drug AEs is obtained.

While package inserts contain the AE information for the patients, the Summaries of product characteristics is the information for the doctors, which is available in the European Union)^96^. Since the Summaries of product characteristics is not available in China, we did not use such information. In the future, we will consider the collection of the Summaries of product characteristics in Europe and use them for our class level effect drug adverse event studies.

FAERS contains publically available data of over 2 million drug AE case reports^97^. Compared to package insert information, the FARES data were spontaneously reported by customers, distributor or health professionals, etc., and can be registered by arbitrary names, including trade names, abbreviations, and even typographical errors. Therefore, the FAERS data can be quite noisy and difficult to analyze. For Such FAERS data analysis, we envision that the conventional drug-AE correlation tests (i.e., PRR, Chi-squared, and minimal case reports) can be performed first, followed by a PCR analysis.

## Conclusion

In this study, we systematically collected cardiovascular drugs (CVDs) used in China and CVD-associated AEs from package insert documents, generated the OCVDAE ontology by reusing terms from the OAE and NDF-RT and adding CVD-AE associations, and systematically analyzed drug class effects on AEs based on OCVDAE knowledge. For the class effect analysis, we for the first time derived a PCR score based on the class effect definition, and calculated and applied PCR scores for heatmap visualization and identification of drug class effects. We also modified classical AE tests to generate the methods of class level PRR (C-PRR), Chi-square (C-χ^2^), minimal number of drugs in a drug class for a specific AE (or AE class). These methods were used to identify statistically significant class effects at the drug ingredient class level and mechanisms of action class level. The usage of our new methods also identified many scientific insights, facilitating knowledge integration and exploration.

## Electronic supplementary material

Supplemental Tables S1 and S2

Supplemental File F1
