# Supplementary material for: Ontology-based systematical representation and drug class effect analysis of package insert-reported adverse events associated with cardiovascular drugs used in China
Source: Sci Rep. 2017 Oct 23;7:13819. doi: 10.1038/s41598-017-12580-4 (PMC5653862; doi:10.1038/s41598-017-12580-4)
Supplement: Supplementary file 1 — Supplemental Tables S1 and S2 [file 41598_2017_12580_MOESM1_ESM.pdf]

Ontology-based systematical representation and drug class effect analysis of package insert-reported adverse events associated with cardiovascular drugs used in China

Liwei Wang[1,2,*], Mei Li[3], Jiangan Xie[4,5], Yuying Cao[1], Hongfang Liu[2], Yongqun He[4,5*]

1. Department of Medical Informatics, School of Public Health, Jilin University, Changchun 130021, China
2. Department of Health Sciences Research, Mayo Clinic, Rochester, MN 55901, USA
3. Library, Hebei Medical University, Shijiazhuang, Hebei Province 050017, China
4. Unit for Laboratory Animal Medicine and Department of Microbiology and Immunology, University of Michigan Medical School, Ann Arbor, MI 48109, USA
5. School of Bioinformatics, Chongqing University of Posts and Telecommunications, Chongqing, 400065, China
6. Center for Computational Medicine and Bioinformatics, University of Michigan Medical School, Ann Arbor, MI 48109, USA

*Corresponding author: wang.liwei@mayo.edu
*Corresponding author: yongqunh@umich.edu

Summary of Supplemental Materials:

Page 2 Supplemental Table S1. CVD class effect based on 36 CVD ingredient classification.

Page 3 Supplemental Table S2. The numbers of drugs and ingredients corresponding to different Mechanisms of Action (MoA) classes.

Supplemental Table S1. CVD class effect based on 36 CVD ingredient classification

Ingredient class	AE	C-PRR	χ^2	PCR
Fatty Acids (including 3 specific drugs: Atorvastatin, Fluvastatin, Fenofibrate)	insomnia AE	2.88	5.22	1
	abdominal pain AE	3.00	5.54	1
	transaminase level increased AE	9.86	19.41	1
	myopathy AE	34.50	41.95	1
	myalgia AE	3.45	6.67	1
	blood creatine phosphokinase level increased AE	9.86	19.41	1
	myositis AE	34.5	41.95	1
	rhabdomyolysis AE	23.00	34.43	1
Glycosides (including 3 ingredients: digoxin, deslanoside, and adenosine)	vision blurred AE	5.31	10.96	1

Note: Each of the three drugs is statistically significantly assocated with each of AEs, which is the meaning of the class effect.

Supplemental Table S2. The numbers of drugs and ingredients corresponding to different Mechanisms of Action (MoA) classes

MoA classes	No. of drugs	No. of ingredients
Cellular or Molecular Interactions [MoA]	166	65
Biological Macromolecular Activity [MoA]	1	1
Enzyme Interactions [MoA]	33	15
Enzyme Inhibitors [MoA]	31	14
Nucleic Acid Synthesis Inhibitors [MoA]	4	2
Nucleoside Synthesis Inhibitors [MoA]	4	2
Phosphodiesterase Inhibitors [MoA]	1	1
Protease Inhibitors [MoA]	11	4
Angiotensin-converting Enzyme Inhibitors [MoA]	11	4
Sterol Synthesis Inhibitors [MoA]	11	5
Cholesterol Synthesis Inhibitors [MoA]	11	5
Physiochemical Activity [MoA]	19	3
Receptor Interactions [MoA]	97	39
Active Transporter Interactions [MoA]	8	4
Neurotransmitter Transporter Interactions [MoA]	3	1
Norepinephrine Transporter Interactions [MoA]	3	1
Small Ion Transport Pump Interactions [MoA]	2	2
G-Protein-linked Receptor Interactions [MoA]	32	20
Adrenergic Receptor Interactions [MoA]	25	16
Adrenergic Agonists [MoA]	13	8
Adrenergic alpha-Agonists [MoA]	4	2
Adrenergic Antagonists [MoA]	10	6
Adrenergic alpha-Antagonists [MoA]	4	2
Adrenergic beta-Agonists [MoA]	3	1
Ion Channel Interactions [MoA]	57	17
Calcium Channel Interactions [MoA]	38	10
Calcium Channel Antagonists [MoA]	37	9
